# Should Animal Welfare Regulations Be More Restrictive? A Case Study in Eight European Union Countries

**DOI:** 10.3390/ani9040195

**Published:** 2019-04-25

**Authors:** Niloofar Pejman, Zein Kallas, Antoni Dalmau, Antonio Velarde

**Affiliations:** 1 Institute for Research in Sustainability Science and Technology (IS-UPC), Polytechnic University of Catalonia, 08034 Barcelona, Spain; 2 Centre for Agro-food Economy and Development, CREDA-UPC-IRTA, 08860 Castelldefels, Spain; 3 Institute of Agrifood Research and Technology—IRTA, Animal Welfare Subprogram, 17121 Monells, Spain; antoni.dalmau@irta.cat (A.D.); antonio.velarde@irta.cat (A.V.)

**Keywords:** animal welfare, citizens, consumers, EU

## Abstract

**Simple Summary:**

Intensive animal production systems are compromising current animal welfare standards. European societies’ growing concerns regarding how animals are raised have resulted in continuous European Union (EU) policy reforms that have banned certain intensive farming methods. We investigated whether EU respondents, differentiated by their roles as citizens and consumers, believe that the current regulations on animal welfare should be more restrictive. Data were collected using a survey approach implemented in eight European countries (Spain, the United Kingdom, Poland, Greece, Lithuania, Romania, Italy, and Sweden) with a sample of 3860 respondents. The results show that women citizens are more concerned with animal welfare and are prone to accept more restrictive regulations. Respondents from northern European countries (Poland and Sweden) are willing to accept regulations that are more restrictive than the current minimum standards than respondents from southern countries (Spain and Italy). Our results suggest that increasing knowledge of animal welfare is related to effective information campaigns that use the Internet to endorse the current animal welfare legislation.

**Abstract:**

Increasingly, intensive livestock production systems have increased societal concern regarding the current animal welfare standards. We investigated whether individuals in their roles as consumers and citizens believe that the current European regulations regarding animal welfare should be more restrictive. Factors affecting this decision were assessed by analyzing respondents’ understanding of animal welfare-related issues, their subjective and objective knowledge levels, the credibility they assign to different information sources, their perceptions toward the current restrictiveness of animal welfare standards, and their socioeconomic characteristics. Data were collected using a semi-structured questionnaire distributed in eight European Union (EU) countries (Spain, the United Kingdom, Poland, Greece, Lithuania, Romania, Italy, and Sweden) with 3860 total responses. The results showed that consumers are more reluctant to adopt more restrictive regulations than respondents in the role of citizens. Respondents from northern European countries (Poland and Sweden) are more likely to support regulations that are more restrictive than the current minimum requirements than respondents from southern countries (Spain and Italy). Women were found to be more concerned with the welfare of pigs and laying hens—lending credibility to the Internet as an information source—and were more likely to support more restrictive animal welfare legislation.

## 1. Introduction

The growing demand for meat products is associated with the increasing human population [1], and income growth [2] has led to an increasing level of animal production [3] and intensive production practices. This production environment has raised societal concerns regarding the current animal welfare standards [4]. The economics-driven perspective on animal welfare depends on understanding the opinions and priorities of the up-stream (supplier and manufacturer) and down-stream (retailers and consumers) stakeholders and policymakers. However, animal welfare standards should guarantee animals certain natural conditions, such as freedom from pain and injury, freedom from hunger and thirst, freedom from discomfort, freedom from fear and distress, freedom to express normal behavior, and human slaughtering [5]. In all cases, the relative importance and relevance of each standard vary, complicating the ability to obtain a consensus regarding what defines adequate animal welfare [6,7].

Animal welfare also includes the quality attributes of animal products, such as meat safety [8], meat quality [9], and sensory factors [10]. Animal welfare can be perceived as an ethical issue that is associated with a set of moral principles related to the right of animals to live. Understating the relative importance of these ethical issues with respect to perceptions of animal welfare may help policymakers to define the moral imperatives to which animal production systems are held by consumers and other stakeholders [11]. The consideration of animal rights is related to individual behaviors and attitudes toward animal production systems [12].

Concerns about animal welfare are also related to an individual’s information level. Lack of knowledge about animal welfare has led to a gap between attitude and behavior [13]. Consumers are, in general, unaware about welfare issues at the farming level [14,15]. However, it is not clear whether consumers are willfully ignorant regarding how animal are raised and thus only focus on other products aspects [16,17,18], or whether they are simply poorly informed about the production process. Trust in information provides important context regarding the conditions in which animal-based foods are produced. The level of trust is associated with the reliability of information sources and certified products related to animal welfare. Currently, European Union (EU) consumers are demanding that food labels be more informative about the methods used for food production. However, they only trust information received from food experts, consumer organizations, and food authorities [19].

Several studies showed that concerns regarding animal welfare are related to animal species [20]. Considerable concern has been found, in general, with respect to pig production systems [21], broilers [22], and laying hens [23]. By the term “broilers”, we refer to *Gallus gallus domesticus*, which is any chicken that is bred and raised for meat production. Consumers are also concerned about laboratory animals used in research, such as rodents and rabbits, and those used in teaching and experiments related to medical issues. The relatively low animal welfare standards may be justified by the social benefits of permitting such animal uses [24]. Many consumers may agree to using animals to support medical issues, whereas they are much more concerned about using animals for developing secondary products, such as cosmetics and furs.

In this environment, animal welfare and its importance within the food system is becoming a prominent and sensitive political issue. EU policymakers are continuously reforming, defining, approving, implementing, and monitoring more restrictive regulations driven by social changes that go beyond the current minimum requirements of animal welfare. EU authorities are responsible for ensuring that such regulations are properly implemented and enforced at all stages of a farm animal’s life—on the farm, during transport, and during slaughtering. The acceptance of these regulatory changes is differentiated by individuals’ roles, perceptions, and attitudes toward animal welfare [25]. Perceptions and attitudes can differ depending on what people think in their different roles as citizens and how they behave as consumers [26,27,28]. Consumers express values and interests related to the process of purchasing, preparing, and consuming animal-based products. These issues for citizens, including vegetarians and vegans, are associated with the organization of society and political issues that may not be influenced by purchasing behavior [29]. Consumers tend to respond to economic incentives with individualistic and materialistic concerns by maximizing their utility and thus rationally choosing products. However, a citizen’s point of view is not necessarily based on economic interest, but can be based on other sets of values more related to altruistic concerns, adopting a holistic approach to society [30]. Whereas citizens and consumers are both concerned about intensive production systems, a discrepancy between their attitudes toward animal welfare has been identified [13]. On the basis of the abovementioned discussion, understanding individuals’ definitions of the animal welfare concept and the factors affecting perception is important. Animal welfare can be affected by individual socio-demographic variables [31], product and animal species [20,32], countries and cultures [33,34], ethical beliefs and opinions, citizens’ and consumers’ perspectives [35,36], and knowledge level [37].

**Figure 1:**
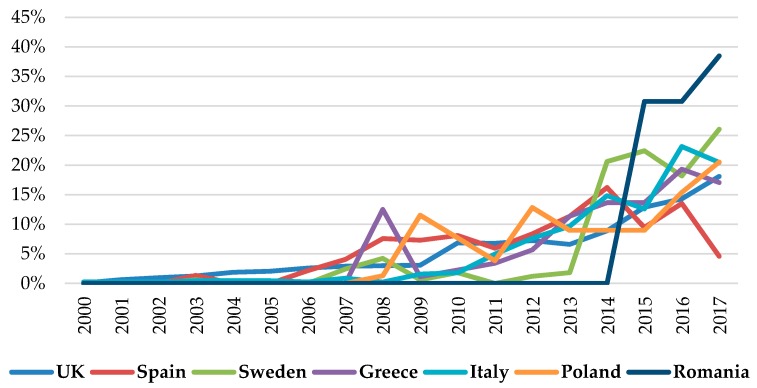
Percentage of annual increase of new products with animal welfare claims in several European Union (EU) countries (own elaboration, from Global New Product Database (GNPD)).

Several studies have shown that animal welfare attitudes can vary across countries and cultures. Animal welfare attitudes can be related to economic development and the modernization of animal farming at the country level [38]. Consumers from southern European countries compared with those from northern countries and the United Kingdom show a higher willingness to pay a premium for products produced under stricter animal welfare standards [39]. Consumers in Sweden and the United Kingdom trust animal production systems that ensure animal welfare standards jointly with public institution interventions [39]. Piglet castration is perceived differently across Europe as an animal welfare issue [40]. While consumers in the United Kingdom agree that pig castration should be banned, the issue is less salient to Spanish consumers [40]. An example of the importance of culture and animal welfare preference is reflected in the number of new animal food products displaying animal welfare claims that have been introduced into the marketplace across the EU. According to the Global New Product Database (GNPD), the number of newly launched meat products advertising animal welfare claims (Figure 1) significantly increased since 2000 to 2017, highlighting the relevance of certain animal welfare attributes in consumers’ purchasing decisions.

In this context, the main objective of this study was twofold: (1) to analyze whether consumers and citizens believe that the current European regulations regarding animal welfare should be more restrictive; and (2) to analyze the determinant factors affecting this decision by analyzing respondents’ understanding of animal welfare-related issues, their subjective and objective knowledge levels, the credibility they assign to the different information sources, their perceptions towards the current level of animal welfare standards, their concerns regarding animal welfare of specific animal species, and their socio-economic characteristics. To achieve the abovementioned objectives, we followed a methodological approach based on face-to-face interviews in eight EU countries (Greece, Italy, Poland, Romania, Spain, Sweden, Lithuania, and the United Kingdom) by surveying 3860 citizens and consumers with approximately 240 respondents organized by group and country.

## 2. Materials and Methods

### 2.1. Data Collection and Sample Size

Data were collected during January–February 2014 using a semi-structured questionnaire distributed in eight European countries (Spain, the United Kingdom, Poland, Greece, Lithuania, Romania, Italy, and Sweden) with different socio-economic and cultural characteristics to determine attitudes toward animal welfare.

Respondents were randomly selected and interviewed in person on different days of the week in different places to ensure the high heterogeneity of the participants. A quota sampling approach was selected. The criteria used to establish the sampling quotas were the following: sex, age, and residence in rural and urban regions in northern, central, and southern locations in each country. An additional stratifying criterion was applied to the respondent profiles to ensure an even representation of consumers and citizens. A total of 96 categories was determined (2 sexes × 4 ages ranges × 2 areas × 3 regions × 2 respondent profiles). Sample quotas were assigned proportionately to the target population (by country) in each quota. Once quotas were calculated for each category, random routes were established to determine the places from which an effective sample could be extracted [41].

To differentiate consumers from citizens, the former was represented by respondents over 18 years of age who are in part or totally responsible for purchasing food and beverages for the household and had purchased and consumed meat products in the last week. In this case, respondents were instructed to complete the survey from the perspective of a consumer of animal products, highlighting their preferences as an individual. The latter was represented by respondents over 18 years of age, including non-consumers of meat products (vegetarians, vegans, etc.). In this case, respondents were asked to consider themselves as members of a society with current values and principles.

The countries were selected according to their different geographical and marketing contexts within Europe with a priori identified distinctive patterns of attitudes, knowledge, and behavior toward animal welfare. The selected countries can be grouped into three subsets a priori based on location: Mediterranean European countries (Greece, Italy, and Spain), Central European countries (Romania, Poland, and Lithuania), and Northern European countries (United Kingdom and Sweden). These countries also exhibit highly heterogeneous socio-economic characteristics that have intensified since the global financial crisis in 2008, reflecting income inequality, labor market and sex gaps, changing unemployment rates, and immigration [42]. The presence of disparities in these socio-economic indicators varies from country to country, even when analyzing relatively similar European countries [43].

The questionnaire was divided into several parts addressing different aspects related to our objectives. The questionnaires distributed to the consumer and citizen groups are provided in Supplementary Materials. The questionnaire was approved by the ethics committee of the Centre for Agro-food Economy and Development of the Polytechnic University of Catalonia (Castelldefels, Spain) and conducted according to the relevant ethical principles, taking specific care to protect personal information according to European General Data Protection Regulation No. 2016/679. Respondents received an explanation of the objective of the study, emphasizing that the information requested would be exclusively used for research and that confidentiality is absolutely guaranteed. Respondents were informed that their participation is voluntary and that they were randomly selected to participate in the study. The template of the consent form can be consulted in the supplementary material (S1). Table 1 provides a summary of the main socio-economic variables of the samples across countries and groups.

### 2.2. Respondents’ Opinions Regarding Whether Animal Welfare Regulations Should Be More Restrictive?

We aimed to analyze factors affecting respondents’ opinions regarding whether or not animal welfare regulations should be more restrictive. Respondents were directly asked if animal welfare regulations should be more restrictive in their countries and were asked to respond with a yes or no answer. For this reason, a binomial logistic regression (logit model) was selected as the best fitting model to describe the relationship between this binary dependent variable and a set of independent variables. The logit model analyzes the probability that an event has success in the response variable (Y = 1) as a linear function of independent variables. In our case, the response variable (Y) has a value of 1 if a respondent answers “yes” for more restrictive animal welfare regulations and a value of 0 if a respondent answers “no” for more restrictive animal welfare regulations. The independent variables were those previously noted as potentially relevant factors and were presented according to the following categories:

(1) Socio-economic variables previously presented in Table 1;
(2) The understanding of the animal welfare concept;
(3) Subjective knowledge level regarding animal welfare;
(4) Objective knowledge level regarding animal welfare;
(5) The credibility of the information source;
(6) Animal welfare concerns for specific animal species;
(7) Perception of the current level of animal welfare standards;
(8) Respondent role (i.e., consumer or citizen); and
(9) Country of residency.

In this case, the logit of this probability $\left(P_{i}\right)$ of answering “Yes” for more restrictive animal welfare regulations is expressed as a function:

(1) $$\ln\left(\frac{P_{i}}{1-P_{i}}\right)=X_{i}^{\prime }\beta^{\prime },$$

where $X_{i}^{\prime }=(1,\;X_{1i},X_{2i}\ldots ,X_{ki})$ represents the (k) independent variables of respondent i and $\beta^{\prime }=(\beta_{0},\;\beta_{1},\;\beta_{2}\;\ldots ,\;\beta_{k})$ is the vector of the coefficients to be estimated through the regression. This logistic regression is posteriorly interpreted by calculating the values of the odds ratios (ORs) for each variable $\left({\mathrm{OR}}_{i}=e^{\beta_{i}}\right)$, which represents the modification that occurs in the response variable for each one-unit change in the independent variable. In other words, it quantifies the increase or decrease in the probability of answering “Yes” for more restrictive animal welfare regulations when the independent variable increases by one unit. For the estimation procedure, the maximum likelihood (ML) criteria and the stepwise method were used for the selection of the independent variables, as they were the best choices in our case. The Wald index was used for each variable’s statistical significance at a 95% confidence level, and the Hosmer and Lemeshow goodness-of-fit test was used to determine the goodness of fit of the model. More detailed information about this regression technique can be found in Paul et al. [44].

#### 2.2.1. Definition of Animal Welfare

The definition of animal welfare it is not only related to the state of the animal’s body, but also to ethical aspects, its feelings, and the living environment. To clarify the meaning of animal welfare, an open question was introduced in the survey to collect respondents’ opinions on this issue. These primary data were analyzed using conventional qualitative content analysis, which provides insight into the interpretation of the meaning of the term from the content of the data by identifying specific categories that refer to different concepts of animal welfare. From the open answer responses, several aspects regarding the perception of animal welfare were identified a posteriori.

To quantify respondents’ understanding of the animal welfare concept, they were asked about their level of agreement with several statements on animal welfare using an 11-point Likert-type scale ranging from 0 (absolutely disagree) to 10 (absolutely agree). The statements on animal welfare were as follows:

(1) Do you agree that animals should be used for work?
(2) Do you agree with using animals for entertainment or sports?
(3) Do you agree with keeping animals for the production of food?
(4) Do you agree with rearing animals for the production of fur?
(5) Do you agree with killing animals when they are seriously injured or ill?
(6) Do you agree with observing animal behavior in an experiment?
(7) Do you agree that medical experiments should be able to use animals to improve human health?
(8) Do you agree with testing cosmetics or household products on animals?
(9) Do you agree with improving animals’ health or increasing their disease resistance via genetic changes?
(10) Do you agree with inflicting pain or injury on animals as part of cultural traditions?

#### 2.2.2. Perceived Subjective and Objective Knowledge Level Regarding Animal Welfare

The study of knowledge level was differentiated between what respondents believe they know (subjective knowledge level) and what they objectively know (objective knowledge level). To analyze both knowledge types, we referred to their subjective experience and objective measurement. Thus, respondents were asked to respond about their perceived knowledge level (subjective) via an 11-point Likert-type scale ranging from 0 (participants do not have any knowledge) to 10 (participants have absolute knowledge). Respondent objective knowledge level was measured by asking respondents to identify eight issues currently regulated in a common policy framework at the EU level from a group of 13 proposed statements about different aspects of animal welfare. For each respondent, an index that counted the correct classification of the aforementioned statements was created. This index ranged from 1 (if a respondent correctly identified only one issue) to 13 (if a respondent correctly identified all the issues). The presentation of the different issues was randomized to mitigate any order bias. The issues presented were the following:

(1) Space allowance per animal in relation to the animal’s weight;
(2) Age at and method of castration of animals;
(3) Limits to the use of cages and ties on animals;
(4) The obligation with respect to certain species to use straw as bedding material or environmental enrichment material;
(5) Animals that are not to be transported;
(6) The obligation to stun animals before slaughtering;
(7) The obligation to feed animals after a certain number of hours at the slaughterhouse;
(8) The obligation to use showers in cases of heat stress (not regulated);
(9) The obligation to have background music in farmyards (not regulated);
(10) The obligation to limit groups of animals to four individuals (not regulated);
(11) The obligation to have available water for animals that are transported, whatever the duration of transport (not regulated);
(12) The obligation to give animals space for resting before slaughter;
(13) Limits to the number of animals per drinking trough in a pen (not regulated).

To compare subjective and objective knowledge levels, both estimated indexes were recalculated in percentage terms. Both types of knowledge levels were related to respondents’ perceptions about the amount of animal welfare information they receive. Respondents were asked if the information they receive in relation to animal welfare is sufficient using an 11-point Likert-type scale ranging from 0 (the information is insufficient) to 10 (the information is sufficient).

#### 2.2.3. Credibility of Information Sources Regarding Animal Welfare

Respondents were asked about their opinions regarding the credibility of the different information sources (n) using an 11-point Likert-type scale ranging from 0 (not credible at all) to 10 (totally credible). The information sources analyzed were the following:

(1) News from television (TV) and radio;
(2) Advertisements from TV and radio;
(3) Specific programs/radio or TV documentaries;
(4) Generalist newspapers;
(5) Specialized magazines;
(6) Books;
(7) Informative brochures;
(8) Formative sessions;
(9) Labels of products;
(10) Communication campaigns of private companies;
(11) Governmental programs;
(12) Generalist websites on the Internet; and
(13) Specialized websites on the Internet.

#### 2.2.4. Perception of Current Level of Animal Welfare Regulations and Concerns Regarding Specific Animal Species

Perceptions of the current level of animal welfare standards in each country may play a relevant role in affecting respondents’ opinions regarding whether more restrictive legislation is warranted. In Italy, Spain, and Poland, the legislation is essentially the same because it originated from the adoption of the same European Commission (EC) directives, whereas in Sweden and the United Kingdom, the legislation on animal welfare includes specific and restrictive national rules, most of which were already in force before the adoption of EC directives. Animal welfare concern in Spain is considered an important issue, but such concern is still lower in Spain than in the observed northern European countries [27]. The Swedish legislation, as well as that of the United Kingdom, takes an individual-focused approach to the welfare of animals. Here, respondents were asked about their perceptions about the current level of animal welfare (p) using an 11-point Likert-type scale ranging from 0 (very low) to 10 (very high).

The relative importance of animal welfare concerns within specific animal production systems was also elicited. Respondents were asked about their concerns regarding animal welfare depending on the animal species using an 11-point Likert-type scale ranging from 0 (not concerned at all) to 10 (I am completely concerned). The following animal production systems were included: laying hens, milk cows, cows for meat, goats for milk/meat, broilers for meat, rabbits for meat, pigs for meat, sheep for milk/meat, and laboratory animals.

## 3. Results and Discussions

### 3.1. Animal Welfare Understanding of Citizens and Consumers

**Figure 2:**
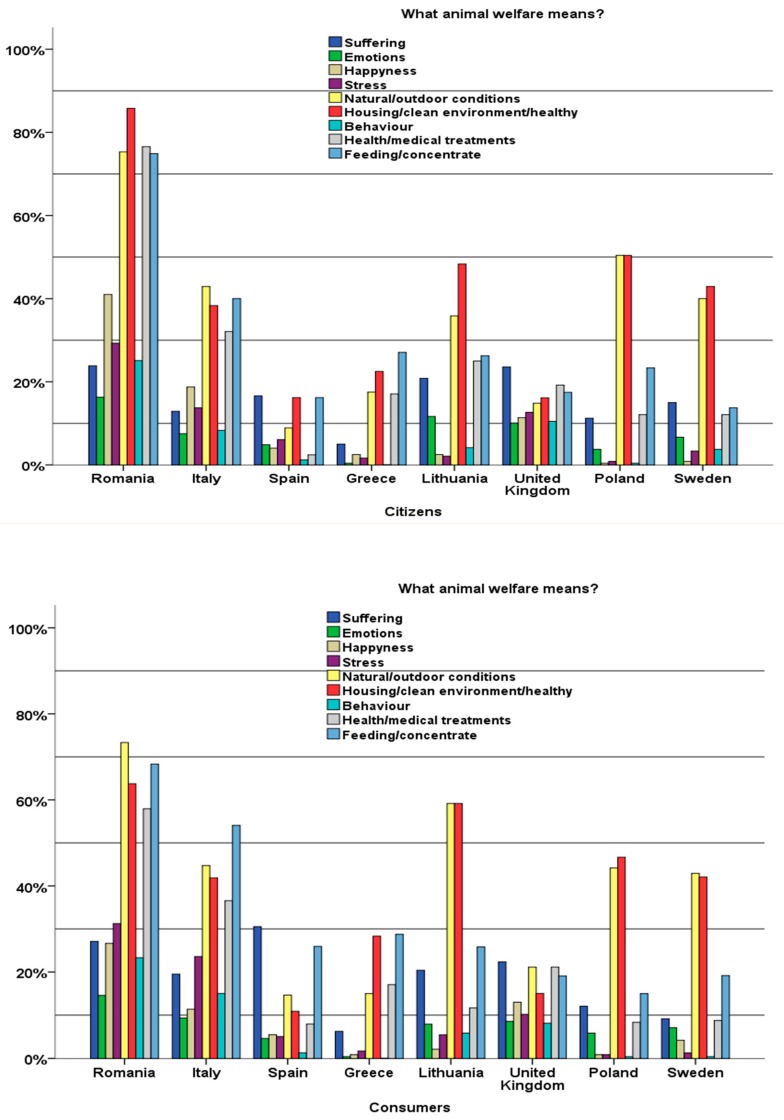
Animal welfare understanding of citizens and consumers.

From the qualitative content analysis, the categories extracted from the open question regarding the meaning of the concept of animal welfare were as follows: suffering, emotions, happiness, stress, natural/outdoor conditions, housing/clean environment/health, behavior, health/medical treatments, and feeding/concentrate. The categories of perceived animal welfare-related issues obtained in this study are in accordance with other findings in the literature regarding public concern about animal welfare. Lassen et al. [45] showed that consumers and citizens tend to understand the definition of animal welfare in terms of housing, outdoor conditions, behavior, and medical treatment. Frewer et al. [27] showed that consumers are concerned about animal welfare with respect to issues of animal health and living environment. The results from European populations showed that predominant concerns are related to issues of natural environments [46], animal suffering [47], and animal well-being [45]. Our results (Figure 2) showed a high level of variation with respect to the understanding of the animal welfare concept across countries and respondent profiles. Some trends were elicited. Focusing on the consumer group, animal welfare was perceived to be more related to natural and outdoor conditions and to clean and healthy housing environments. These were the issues that were most often raised by consumers in Sweden, Poland, Lithuania, and Romania. However, consumers in Italy and Greece highlighted good feeding as the most important aspect of animal welfare. The results show that consumers in the United Kingdom do not prioritize any specific issues, as almost all factors were noted with equal importance; however, citizens in the United Kingdom assigned the highest overall score to the avoidance of pain and suffering. Finally, consumers in Spain highlighted the relevance of avoiding suffering as an important aspect of animal welfare. Citizens in Romania, Lithuania, Italy, Poland, and Sweden attributed the highest importance to aspects related to natural living conditions and clean environment. Respondents from the northern European countries assigned lower values to the aspects of feeding, pain, and healthiness in animal welfare compared with those from other regions.

Independent of the identified differences between the consumer and citizen points of view, content was analyzed in each country for the pooled sample. The results showed that respondents in Romania, Lithuania, Poland, and Sweden are more interested in the “natural conditions and clean environment” of animal rearing. These results highlight that animal welfare concerns in certain countries (Lithuania, Poland, and Sweden) include more than just feeding conditions, medical treatment, and animal stress, being more related to the natural conditions of living with outdoor access. The Mediterranean countries (Italy, Greece, and Spain) assign more importance to the suitable and natural feeding of animals. However, avoiding pain and suffering was the most important aspect reported in Spain. This could be because of the several cultural activities in Spain involving animals, such as quail catapulting, horse wrestling, goose decapitating, throwing a goat off a building, donkey stoning, and setting a bull’s face on fire.

### 3.2. Perceived Subjective and Objective Knowledge Level Regarding Animal Welfare

The results in Table 2 show that consumers and citizens in Romania, Italy, Spain, and Greece have a lower subjective knowledge level of animal welfare (below 50%) compared with those in Lithuania, the United Kingdom, Poland, and Sweden, whose values were higher than 50%. The results also show a low level of objective knowledge in all countries, with the number of correct answers being below 50%. For each type of respondent and country, we compared the subjective and objective knowledge levels. In the majority of the results, significant differences were obtained, showing that respondents tended to exhibit higher subjective knowledge than what they know objectively. If we analyzed the differences across countries, there was a clear differentiation between two groups of countries. The first group (Romania, Italy, Spain, and Greece) exhibited a low discrepancy level (i.e., the difference between the subjective and objective knowledge levels) compared with the other group (Lithuania, the United Kingdom, Poland, and Sweden), whose respondents believed that they know a lot more than they do in reality. This higher discrepancy level was identified in countries whose respondents selected a higher agreement level with the assertion that they receive sufficient information regarding animal welfare. This could have created an artificial confidence that led respondents to believe that they know more than they do. Any information provided regarding animal welfare appears to not have been completely absorbed. These results also highlight the positive correlation between the sufficient information level and respondents’ objective and subjective knowledge. When agreement with sufficient information increased, exhibited knowledge increased as well. This helps to explain why respondents in Italy, Spain, and Greece exhibited a lower knowledge level compared with those in Lithuania, the United Kingdom, Poland, and Sweden.

The results also show that when respondents’ agreement regarding sufficient information was above average (i.e., >5.5 points on an 11-point scale), a non-significant association was found, as was the case with Sweden and Poland. These results suggest that policies based on increasing information campaigns about animal welfare as a tool to provide consumers and citizens with sufficient information could positively impact their knowledge level, but only if such campaigns are conducted with adequate intensity to be efficient. Policy tools should identify the optimum effort required for such information campaigns, because a higher level of information does not necessarily translate to retained knowledge and may result in higher discrepancy levels.

Providing consumers and citizens with sufficient information about animal welfare may effectively improve their knowledge. Therefore, identifying which information sources are the most effective is important. In this context, understanding respondents’ perception regarding the credibility of information sources is highly relevant for the development of effective information campaigns regarding animal welfare.

### 3.3. Credibility of Information Sources

The credibility of information resources was assessed using principal component analysis (PCA). The results show the presence of four main factors with high goodness of fit, explaining 58.53% of the total explained variance, a Kaiser–Meyer–Olkin measure of about 0.820, and a very good significance level with respect to Bartlett’s Test of Sphericity (0.000). Factor 1 (17.55% of explained variance) was called “specialized written media”, as it encompasses information sources from books, specialized magazines, formative sessions, and informative brochures. Factor 2 (16.47% of explained variance) was categorized as traditional media and contained the following information sources: news from TV and radio, advertisements from TV and radio, specific programs/radio or TV documentaries, and generalist newspapers. Factor 3 (13.33% of explained variance) was defined as market information, including information from the labels of products, communication campaigns of private companies, and governmental programs. Factor 4 (11.18% of explained variance) was labeled “Internet” and contained specialized and generalist websites on the Internet.

The relation between the credibility of the information using PCA and the level of objective and subjective knowledge was analyzed. The results showed that specialized written media are the most important factor that can affect citizens’ and consumers’ knowledge, especially the objective information level. A significant and positive relationship was found for this relationship in almost all countries with the exception of the United Kingdom and Poland. Toma et al. [48] found that access to information has a significant influence regarding attitudes toward and knowledge of animal welfare. In Italy, Spain, and Greece, the results showed that an increase in the credibility of specialized written media was related to an increase in the objective knowledge level. In Lithuania, respondents exhibited higher levels of both objective and subjective knowledge, where specialized written media are the most affordable means of information, as also highlighted in Marcus et al. [49]. This result is also supported by two studies [24,50], which reported that consumers are more affected by public information campaigns based on posters/brochures and labels. The results also showed that Internet websites are relevant as important media, having a major effect on the subjective knowledge level. To summarize in general terms, the results showed that respondents who rely on the Internet exhibit higher subjective information levels than those who rely on specialized media, who display higher objective information levels. On the basis of respondents’ knowledge, traditional media and market information were found to be less effective communication tools.

### 3.4. Animal Welfare Concerns Regarding Different Animal Species

As previously stated, respondents indicated varying levels of concern regarding animal welfare for different animal species. The results in Table 3 show different levels of concern across countries and respondent types. Consumers exhibited greater animal welfare concern than citizens. In this case, consumers exhibited a greater level of concern because they appreciate the quality and safety guaranteed by more restrictive animal welfare standards [21,51]. As noted by Serpell [52], consumers are more utility-oriented and their concerns are not solely motivated by ethical considerations.

Respondents in Spain, Italy, Greece, and Romania exhibited greater concern regarding animal welfare compared with those in the other countries, in particular for pigs for meat, broilers for meat, milk cows, cows for meat, and laying hens. Pork is one of the most produced and consumed meats in the EU [28]. Respondents in Italy, consumers in Spain, and citizens in Sweden showed high levels of concern with respect to broiler production. Respondents in Lithuania and Poland, consumers in Sweden, and citizens in Spain are more concerned with pig production systems. These results are in accordance with the findings of Driscoll et al. [53], which showed that individual attitudes toward animals are highly related to animal species.

### 3.5. Respondents’ Opinions on Whether Animal Welfare Regulations Should Be More Restrictive

Following the methodological approach, a logit model was applied to analyze factors affecting respondents’ decision to support more restrictive regulations of animal welfare. The descriptive results of the dependent variable (Table 4) show two distinct opinions: consumers and citizens in Romania, Italy, Spain, and the United Kingdom exhibited reluctance to support more restrictive regulations regarding animal welfare; whereas Poland, Sweden, Lithuania, and Greece showed greater interest in more restrictive regulations. Sweden has the most advanced legislation related to animal protection, and Swedish consumers were found to be less worried about animal welfare, exhibiting a higher trust level in their animal production systems and regulations [19]. However, the rejection of more restrictive regulations regarding animal welfare could be related to cultural traditions, such as in the case of Spain, where animals play a significant role.

For the logit model estimation, we used the pooled dataset by including a dummy variable for the respondent types, that is, 1 for citizens and 0 for consumers. For each country, a dummy variable was created to include heterogeneity across countries if needed. The goodness of fit was measured by the Hosmer–Lemeshow test, which ensures that all coefficients jointly are different from zero. The results of the logit model are shown in Table 5.

Our results show an acceptable rate (62.1%) of correct prediction representing the probability of accepting more restrictive legislation based on a one-unit change in an independent variable when all other independent variables are kept constant. The respondent type is a relevant variable in explaining the decision to support more restrictive regulations regarding animal welfare. Citizens showed a higher likelihood of accepting more restrictive regulations than consumers. This outcome demonstrates that citizens, even when exhibiting less concern regarding animal welfare, are more likely to agree to more restrictive regulations. Our results suggest that, compared with consumers, a smaller increase in citizens’ concern translates to greater support for restrictive regulations. These results are in line with those of Clark et al. [21], who found that greater concern voiced by citizens indicates that legislative solutions are necessary for ensuring animal welfare standards. Respondents may behave differently in the role of citizen versus consumer by expressing different preferences for animal welfare when interviewed [46]. In this context, the importance of animal welfare for consumers in their purchasing decisions can be related to other attributes, such as price, origin, color, or tenderness, or to other barriers regarding purchasing animal-based products produced with higher animal welfare standards [13,21]. However, in some studies, non-significant differences were elicited [13,54], showing the importance of differentiating between both groups and updating our understanding regarding their perceptions.

Respondents from Poland and Sweden were prone to supporting more restrictive regulations. Respondents who exhibited high subjective information levels were more concerned with the welfare of laying hens, broilers, and pigs, and were more likely to agree with adopting more restrictive animal welfare legislation. Respondents who attributed higher credibility to Internet information showed a higher likelihood of accepting more restrictive regulations. Respondents from Spain and Italy were less likely to accept more restrictive regulations. These results highlight the Spanish opinion regarding animal welfare legislation. According the Eurobarometer survey [38], 60% of respondents believed that welfare protection had improved over the last 10 years, so there is probably no need for additional restrictive regulations. Compared with those from other European countries, respondents from northern European countries showed the greatest concern for animal welfare in farm production systems. Respondents who perceived that the current animal welfare level in their country is high and who agree with using animals for fur and cosmetic production, work, and sports were less likely to accept more restrictive regulations. Finally, men exhibited less interest in adopting more restrictive animal welfare regulations. In general, women are more concerned about this issue and are more likely to support more restrictive regulations regarding animal welfare [55]. Women generally demonstrate more affection toward animals and exhibit a greater preference for more restrictive animal welfare standards [46,56].

## 4. Conclusions

We identified factors affecting consumer and citizen opinions regarding whether animal welfare regulations should be more restrictive in eight EU countries. These factors were categorized into the understanding of animal welfare-related issues, subjective and objective knowledge level regarding animal welfare, the credibility of information sources, the perception of the current level of animal welfare standards in each country, concerns regarding specific animal species, and socio-economic characteristics. Our model showed two clearly differentiated behaviors: respondents in southern EU countries (Italy and Spain) exhibited significant reluctance to the implementation of more restrictive regulation and those in northern EU countries (Poland and Sweden) exhibited the opposite opinions.

The respondent type played a relevant role in explaining the respondents’ preferences for accepting more restrictive regulations beyond the minimum requirements. Respondents in the citizen role showed greater willingness to accept more restrictive regulations compared with those in the consumer role. Respondents with a higher level of subjective knowledge on animal welfare, women, and those who assign high credibility to the Internet as an information source exhibited greater preferences for adopting more restrictive legislation. The results showed that having more concerns regarding pig production systems and laying hens increases the likelihood that a respondent will accept more restrictive regulations on animal welfare. These results are in accordance with the special attention that European authorities are paying to these two types of production systems. In 2013, the EU banned the use of individual sow stalls in pig production, and in 2012, the EU banned the use of conventional cages for laying hen production. Thus, these results highlight the importance of policymakers adopting reforms that are in accordance with societal preference and concerns to create more effective and acceptable animal welfare policies.

Respondents who perceive that the level of current animal welfare standards in their country is high were less likely to accept more restrictive regulations. Our results highlight the need for Mediterranean countries to increase animal welfare knowledge to justify to their citizens the need for increasingly restrictive EU regulations. Thus, information campaigns using the Internet as a credible media source to promote current animal welfare standards can be used to affect public opinion. This study highlights the importance of Internet websites in affecting respondents’ opinions and thus their knowledge, because such sites often play a relevant role in forming the decision to adopt more restrictive regulations. The results should be handled carefully because of the hypothetical nature of our survey.

The hypothetical bias is one of the major drawbacks when analyzing consumers’ perceptions and opinions towards ethical issues such as animal welfare. According to an old saying, “there is a difference between saying and doing”. Respondents tend to behave as they would like to be and not as they really are. In this context, the decision to accept more restrictive animal welfare regulations was not conditioned to any “non-hypothetical” behavior. Thus, further research that accounts for the hypothetical bias by allowing the survey to be consequential is needed. Including market consequences for such a decision such as an increase of the production cost at farm level and a higher price of meat products is of interest.

## Figures and Tables

**Table 1:** Summary of socio-demographic variables of the samples by groups and country (values are in percentage).

Country		Romania		Italy		Spain		Greece		Lithuania		United Kingdom		Poland		Sweden	
Region (%)		P	C	P	C	P	C	P	C	P	C	P	C	P	C	P	C
	North	33.1	33.5	33.8	52.6	35.2	33.6	33.3	33.3	26.7	36.3	40.9	27.9	43.8	78.8	-	15.4
	Center	66.9	66.5	33.8	17.0	31.2	32.4	33.3	33.3	40.0	23.1	46.1	57.9	32.9	20.8	13.3	84.6
	South	-	-	32.5	30.4	33.6	34.0	33.3	33.3	33.3	40.6	13.0	14.2	23.3	0.4	86.7	-
Area (%)	Rural	49.4	50.0	50.8	51.4	53.0	52.3	50.0	50.0	24.2	21.8	43.5	45.3	33.8	55.8	53.8	17.1
	Urban	50.6	50.0	49.2	48.6	47.0	47.7	50.0	50.0	75.8	78.2	56.5	54.7	66.3	44.2	46.3	82.9
Employment situation (%)	Unemployed	1.7	1.7	12.9	6.9	9.3	4.9	19.2	19.2	9.2	13.8	8.3	5.3	2.5	2.1	5.0	4.2
	Self-employed	17.0	16.9	16.7	27.1	5.7	4.5	25.5	21.3	0.4	1.3	6.5	8.5	-	2.5	8.3	5.0
	Salaried	63.0	65.7	18.8	34.4	51.8	77.9	31.0	40.0	55.8	66.3	55.2	57.5	69.2	84.2	64.2	68.3
	Retired	9.4	8.7	5.8	10.9	0.4	6.1	12.6	8.3	0.8	3.3	3.9	2.4	5.8	2.1	0.8	-
	Student	4.7	2.9	42.1	9.3	32.8	3.7	7.9	7.5	32.5	14.6	17.8	18.6	22.5	7.9	19.2	20.8
	Housewife	4.3	4.1	3.3	10.9	-	2.9	3.8	3.3	1.3	0.8	0.4	2.8	-	1.3	2.5	1.7
Gender (%)	Female	66.1	56.2	45.4	70.0	57.9	62.7	62.9	62.9	80.0	82.9	50.0	59.5	72.9	67.1	74.6	77.1
	Male	33.9	43.8	54.6	30.0	42.1	37.3	37.1	37.1	20.0	17.1	50.0	40.5	27.1	32.9	25.4	22.9
Age categories (%)	18–30	33.1	19.8	60.8	20.6	46.2	25.0	26.7	29.6	67.9	56.3	60.3	56.3	48.3	27.9	49.6	40.4
	31–40	29.3	37.6	18.3	27.9	27.1	34.8	20.8	25.0	16.7	14.6	14.0	15.4	17.9	28.7	19.2	23.8
	41–55	27.6	31.8	13.8	32.8	20.6	24.6	37.5	35.0	11.7	14.6	19.2	20.6	20.4	32.1	29.6	23.8
	>55	10.0	10.7	7.1	18.6	6.1	15.6	15.0	10.4	3.8	14.6	6.6	7.7	13.3	11.3	1.7	12.1
Age (average years)		38.15	39.75	31.87	42.83	33.81	40.29	41.43	40.08	29.72	34.82	32.53	33.36	35.76	39.32	33.36	36.47
Observations by respondent type		239	242	240	247	247	247	240	240	240	240	230	248	240	240	240	240
Sample Size		481		487		494		480		480		478		480		480	
Confidence interval		4.47%		4.44%		4.41%		4.47%		4.47%		4.48%		4.47%		4.47%	

P: citizens; C: consumers.

**Table 2:** Objective and subjective knowledge levels.

		Subjective Knowledge Level	Objective Knowledge Level	Discrepancy Intensity between Knowledge	Sufficient Information Level	Subjective Information Level	Objective Information Level
Romania	P	44.51 ^a,x^	39.26 ^b,x^	5.25	4.66	+ ***	+ ***
	C	45.77 ^a,x^	37.64 ^b,x^	8.06	4.15	+ ***	+ ***
Italy	P	45.29 ^a,x^	43.01 ^a,x^	2.28	3.45	+ ***	
	C	45.75 ^a,x^	41.39 ^b,x^	4.36	3.15	+ ***	
Spain	P	43.19 ^a,x^	37.27 ^b,x^	5.92	3.62	+ ***	
	C	42.48 ^a,x^	33.65 ^b,x^	8.83	3.74	+ ***	
Greece	P	44.04 ^a,x^	41.18 ^b,x^	2.86	2.27	+ ***	+ **
	C	40.83 ^a,x^	37.28 ^a,x^	3.55	2.14	+ ***	+ **
Lithuania	P	62.87 ^a,x^	52.69 ^b,x^	10.18	4.85	+ ***	+ **
	C	49.96 ^a,y^	39.29 ^b,y^	10.67	3.82	+ ***	+ ***
United Kingdom	P	50.52 ^a,x^	38.06 ^b,x^	12.46	4.48	+ ***	+ ***
	C	51.84 ^a,y^	34.10 ^b,x^	17.74	4.19	+ ***	
Poland	P	65.50 ^a,x^	49.74 ^b,x^	15.76	5.71		
	C	59.58 ^a,x^	42.98 ^b,x^	16.60	5.50		
Sweden	P	54.20 ^a,y^	48.58 ^a,y^	5.62	6.27		
	C	60.21 ^a,x^	46.47 ^b,x^	13.74	6.58		

The level of the subjective knowledge is measured in percentage term where 0 indicates very a low knowledge level and 100 a very high knowledge level. The level of the objective knowledge represents the percentage of successful rate of correct answers. The sufficient information level is measured with an 11-point Likert-type scale that ranges from 0 (the information is insufficient) to 10 (the information is sufficient). ^a, b^: statistical difference between the subjective and objective knowledge level (i.e., by row); ^x, y^: statistical difference between citizens and consumers (i.e., by column); ***: significance at 99% level, **: significance at 95% level.

**Table 3:**
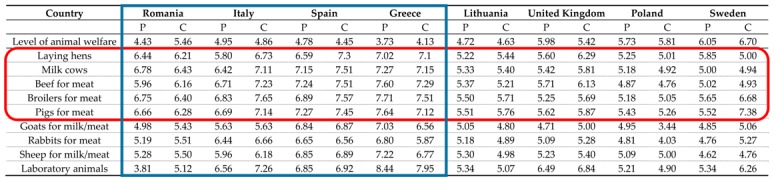
Animal welfare concerns by animal species and respondent type.

P: public (citizens), C (consumers). Animal welfare concerns are measured using an 11-point Likert-type scale ranging from 0 (not concerned at all) to 10 (I am totally concerned).

**Table 4:** Should regulation be more restrictive across countries and respondent type?

		Yes	No
Romania	Citizens	46.40	53.60
	Consumers	43.00	57.00
Italy	Citizens	35.90	64.10
	Consumers	51.80	48.20
Spain	Citizens	32.40	67.60
	Consumers	47.50	52.50
Greece	Citizens	68.80	31.30
	Consumers	67.90	32.10
Lithuania	Citizens	54.00	46.00
	Consumers	42.10	57.90
United Kingdom	Citizens	37.00	63.00
	Consumers	35.20	64.80
Poland	Citizens	52.10	47.90
	Consumers	53.10	46.90
Sweden	Citizens	51.20	48.80
	Consumers	53.10	46.90

**Table 5:** Logit model to analyze factor affecting the agreement with more restrictive regulations.

	B	Sig.	Exp (B)
Type of questionnaire (q)	0.29	0.000	1.33
Sweden (r)	0.20	0.063	1.23
Poland (r)	0.24	0.030	1.27
Subjective information level (l)	0.10	0.001	1.10
Concerns for laying hens/broilers for meat (o)	0.07	0.000	1.07
Credibility of Internet media (factor) (n)	0.06	0.081	1.06
Concerns for pigs animal welfare (o)	0.03	0.036	1.03
Spain (r)	−0.34	0.002	0.71
Italy (r)	−0.18	0.090	0.83
Gender (j)	−0.12	0.090	0.88
Perceived current animal welfare level (p)	−0.09	0.000	0.92
Animal use for fur, work, sport, and cosmetics (k)	−0.04	0.000	0.96
Correct classification	62.1%		
Hosmer and Lemeshow Test (Sig. = 0.12)			

## Supplementary Materials

The following are available online at https://www.mdpi.com/2076-2615/9/4/195/s1, S1: Template of the consent form.

## References

[1] Ballard D., Peterson E., Nadler J., Khardori D. (2015). Antibiotic Use in Animal Feed and Its Impact on Antibiotic Resistance in Human Pathogens. Food Microbiol..

[2] Fukase J., Simons A.M. (2016). Increased pollinator activity in urban gardens with more native flora. Appl. Ecol. Environ. Res..

[3] Rollin B.E. (2005). Reasonable partiality and animal ethics. Ethical Theory Moral Pract..

[4] Webster A.J.F. (2001). Farm Animal Welfare: The Five Freedoms and the Free Market. Vet. J..

[5] Rushen J., Passillé A.M.B.D. (2010). The scientific assessment of the impact of housing on animal welfare: A critical review. Can. J. Anim. Sci..

[6] Mason J., Mendl M. (2007). Why is there no simple way of measuring animal welfare?. Anim. Welf..

[7] Lund V. (2006). Natural living-a precondition for animal welfare in organic farming. Livest. Sci..

[8] Cembalo V., Caracciolo F., Lombardi A., Del Giudice T., Grunert K.G., Cicia G. (2016). Determinants of Individual Attitudes Toward Animal Welfare-Friendly Food Products. J. Agric. Environ. Ethics.

[9] Thorslund C.A., Aaslyng M.D., Lassen J. (2017). Perceived importance and responsibility for market-driven pig welfare: Literature review. Meat Sci..

[10] Beattie V.E., O’Connell N.E., Moss B.W. (2000). Influence of environmental enrichment on the behaviour, performance and meat quality of domestic pigs. Livest. Prod. Sci..

[11] Engster D. (2006). Care Ethics and Animal Welfare. J. Soc. Philos..

[12] Rozin P. (1996). Towards a psychology of food and eating: From motivation to module to model to marker, morality, meaning, and metaphor. Curr. Dir. Psychol. Sci..

[13] Harper G., Henson S. (2001). Consumer Concerns about Animal Welfare and the Impact on Food Choice.

[14] Schröder M.J.A., McEachern M.G. (2004). Consumer value conflicts surrounding ethical food purchase decisions: A focus on animal welfare. Int. J. Consum. Stud..

[15] McEachern M.G., Schróder M.J.A. (2002). The role of livestock production ethics in consumer values towards meat. J. Agric. Environ. Ethics.

[16] Bell E., Norwood F.B., Lusk J.L. (2017). Are consumers wilfully ignorant about animal welfare?. Anim. Welf..

[17] Borrisser-Pairó F. (2016). Prevalence of boar taint in commercial pigs from Spanish farms. Meat Sci..

[18] Heerwagen L.R., Christensen T., Sandøe P. (2013). The prospect of market-driven improvements in animal welfare: Lessons from the case of grass milk in Denmark. Animals.

[19] Kjærnes U., Bock B.B., Roe E., Roex J. Consumption, Distribution and Production of Farm Animal Welfare. https://eprints.soton.ac.uk/72066/1/72066-01.pdf.

[20] Cicia G., Colantuoni F. (2010). Willingness to pay for traceable meat attributes: A meta-analysis. Int. J. Food.

[21] Clark B., Stewart G.B., Panzone L.A., Kyriazakis I., Frewer L.J. (2016). A Systematic Review of Public Attitudes, Perceptions and Behaviours Towards Production Diseases Associated with Farm Animal Welfare. J. Agric. Environ. Ethics.

[22] Broom D.M. (2017). Animal Welfare in the European Union. Eur. Union.

[23] Campbell D.L.M., Hinch G.N., Downing J.A., Lee C. (2017). Outdoor stocking density in free-range laying hens: Effects on behaviour and welfare. Animal.

[24] Carbone L. (2004). What Animals Want: Expertise and Advocacy in Laboratory Animal Welfare Policy.

[25] Verbeke W. (2009). Stakeholder, citizen and consumer interests in farm animal welfare. Anim. Welf..

[26] Te Velde H., Aarts N., Van Workum C. (2002). Dealing with ambivalence: farmers’ and consumers’ perceptions of animal welfare in livestock breeding. J. Agric. Environ. Ethics.

[27] Frewer L.J., Kole A., Van De Kroon S.M.A., De Lauwere C. (2005). Consumer attitudes towards the development of animal-friendly husbandry systems. J. Agric. Environ. Ethics.

[28] Krystallis A., de Barcellos M.D., Kügler J.O., Verbeke W., Grunert K.G. (2009). Attitudes of European citizens towards pig production systems. Livest. Sci..

[29] Korzen S., Lassen J. (2010). Meat in context. On the relation between perceptions and contexts. Appetite.

[30] Berglund C., Matti S. (2006). Citizen and consumer: The dual role of individuals in environmental policy. Environ. Polit..

[31] Bayarri S., Martí M., Carbonell I., Costell E. (2012). Identifying drivers of liking for commercial spreadable cheeses with different fat content. J. Sens. Stud..

[32] Carlsson F., Lagerkvist C.J. (2004). Consumer willingness to pay for farm animal welfare—Transportation of farm animals to slaughter versus the use of mobile abattoirs. Eur. Rev. Agric. Econ..

[33] Bernard J.C., Bernard D.J. (2009). What is it about organic milk? An experimental analysis. Am. J. Agric. Econ..

[34] Carlsson F., Frykblom P., Lagerkvist C.J. (2007). Consumer willingness to pay for farm animal welfare: Mobile abattoirs versus transportation to slaughter. Eur. Rev. Agric. Econ..

[35] Angie A.D., Connelly S., Waples E.P., Kligyte V. (2011). The influence of discrete emotions on judgement and decision-making: A meta-analytic review. Cogn. Emot..

[36] Brunstrom J.M., Shakeshaft N.G., Scott-Samuel N.E. (2008). Measuring ‘expected satiety’ in a range of common foods using a method of constant stimuli. Appetite.

[37] Tonsor G.T., Olynk N.J. (2011). Impacts of Animal Well-Being and Welfare Media on Meat Demand. J. Agric. Econ..

[38] European Commission (2005). Europeans, Science and Technology.

[39] Veissier I., Butterworth A., Bock B., Roe E. (2008). European approaches to ensure good animal welfare. Appl. Anim. Behav. Sci..

[40] Kallas Z. (2013). Effect of tasting and information on consumer opinion about pig castration. Meat Sci..

[41] Kallas Z., Gómez-Limón J.A., Arriaza M. (2007). Are citizens willing to pay for agricultural multifunctionality?. Agric. Econ..

[42] The Organisation for Economic Co-operation and Development (OECD) Understanding the Socio-economic Divide in Europe Centre for Opportunity and Equality. https://www.oecd.org/els/soc/cope-divide-europe-2017-background-report.pdf.

[43] Marcińczak S., Musterd S. (2012). Inequality and Rising Levels of Socio-Economic Segregation: Lessons from a Pan-European Comparative Study.

[44] Paul P., Pennell M.L., Lemeshow S. (2013). Standardizing the power of the Hosmer-Lemeshow goodness of fit test in large data sets. Stat. Med..

[45] Lassen J., Sandøe P., Forkman B. (2006). Happy pigs are dirty!—Conflicting perspectives on animal welfare. Livest. Sci..

[46] Vanhonacker F., Verbeke W., Van Poucke E., Tuyttens F.A.M. (2008). Do citizens and farmers interpret the concept of farm animal welfare differently?. Livest. Sci..

[47] Phillips C.J.C., Wojciechowska J., Meng J., Cross N. (2009). Perceptions of the importance of different welfare issues in livestock production. Animal.

[48] Toma L., Stott A.W., Revoredo-Giha C., Kupiec-Teahan B. (2012). Consumers and animal welfare. A comparison between European Union countries. Appetite.

[49] Marcus B.H., Owen N., Forsyth L.H., Cavill N.A., Fridinger F. (1998). Physical activity interventions using mass media, print media, and information technology. Am. J. Prev. Med..

[50] Whiting T.L. (2003). Foreign animal disease outbreaks, the animal welfare implications for Canada: Risks apparent from international experience. Can. Vet. J..

[51] Boogaard B.K., Oosting S.J., Bock B.B., Wiskerke J.S.C. (2011). The sociocultural sustainability of livestock farming: An inquiry into social perceptions of dairy farming. Animal.

[52] Serpell J.A. Factors Influencing Human Attitudes to Animals and Their Welfare. https://www.researchgate.net/publication/263077760_Factors_Influencing_Human_Attitudes_to_Animals_and_Their_Welfare.

[53] O’Driscoll K., Von Keyserlingk M.A.G., Weary D.M. (2010). Effects of Mixing on Drinking and Competitive Behavior of Dairy Calves. J. Dairy Sci..

[54] Grunert K.G., Sonntag W.I., Glanz-Chanos V., Forum S. (2018). Consumer interest in environmental impact, safety, health and animal welfare aspects of modern pig production: Results of a cross-national choice experiment. Meat Sci..

[55] Cornish A., Raubenheimer D., McGreevy P. (2016). What we know about the public’s level of concern for farm animal welfare in food production in developed countries. Animals.

[56] Lagerkvist C.J., Hess S. (2011). A meta-analysis of consumer willingness to pay for farm animal welfare. Eur. Rev. Agric. Econ..

