# Supplementary material for: Should Animal Welfare Regulations Be More Restrictive? A Case Study in Eight European Union Countries
Source: Animals (Basel). 2019 Apr 25;9(4):195. doi: 10.3390/ani9040195 (PMC6523126; doi:10.3390/ani9040195)
Supplement: Supplementary file 1 [file animals-09-00195-s001.pdf]

CONSENT FORM

You have been **randomly selected** to participate in a study in which you will be asked to answer a questionnaire of about 15 minutes. The questionnaire consists of different sections regarding your attitudes, perceptions and understanding of Animal Welfare (AW) concept, and opinions if the current regulations regarding AW should be more restrictive.

This study is a part of a European research project called Educawel with title *Study on Education and Information Activities on Animal Welfare* that is financed by the European Union- Directorate-General "Health and Consumer Protection" under the grant agreement SANCO/2013/G3/SI2.649393. This project is an academic research and does **not represent any commercial** or **company interest**.

The information requested in the survey will be **exclusively used for research. Its confidentiality is absolutely guaranteed and will never be revealed to third parties**. **It is not necessary to give us information you do not want to provide and you can withdraw from answering at any time you want**.

Thanks in advance for participating to our research

DATE: ______________________2013

Signature

This project has received funding from the European Union DG-SANCO (Directorate-General "Health and Consumer Protection" under grant agreement SANCO/2013/G3/SI2.649393 (project acronym EDUCAWEL Study on Education and Information Activities on Animal Welfare). This project is an academic research and does not represent any commercial or company interest..
